# leeHom: adaptor trimming and merging for Illumina sequencing reads

**DOI:** 10.1093/nar/gku699

**Published:** 2014-08-06

**Authors:** Gabriel Renaud, Udo Stenzel, Janet Kelso

**Affiliations:** Department of Evolutionary Genetics, Max Planck Institute for Evolutionary Anthropology, D-04103 Leipzig, Germany; Department of Evolutionary Genetics, Max Planck Institute for Evolutionary Anthropology, D-04103 Leipzig, Germany; Department of Evolutionary Genetics, Max Planck Institute for Evolutionary Anthropology, D-04103 Leipzig, Germany

## Abstract

The sequencing of libraries containing molecules shorter than the read length, such as in ancient or forensic applications, may result in the production of reads that include the adaptor, and in paired reads that overlap one another. Challenges for the processing of such reads are the accurate identification of the adaptor sequence and accurate reconstruction of the original sequence most likely to have given rise to the observed read(s). We introduce an algorithm that removes the adaptors and reconstructs the original DNA sequences using a Bayesian maximum *a posteriori* probability approach. Our algorithm is faster, and provides a more accurate reconstruction of the original sequence for both simulated and ancient DNA data sets, than other approaches. leeHom is released under the GPLv3 and is freely available from: https://bioinf.eva.mpg.de/leehom/

## INTRODUCTION

Recent improvements have increased the efficiency with which endogenous molecules from ancient samples can be made accessible for sequencing (1). However, the DNA molecules extracted from ancient samples are often short due to the degradation of DNA after the death of the organism and average length rarely exceeds 100 bp (2).

**Figure 1.**
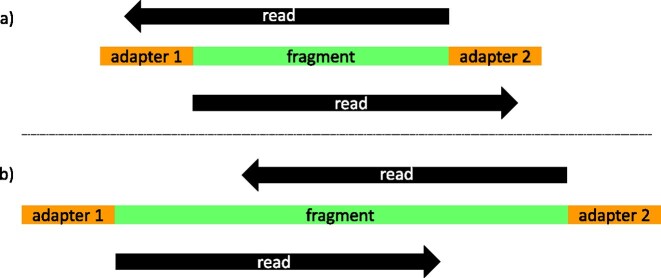
Schematic representation of paired-end sequencing for very short molecules. (a) When the molecule is shorter than the read length, both reads will run into the adaptors and the remaining part will completely overlap. (b) If the sequence is longer but still not longer than twice the read length, adaptor sequences will be absent but a partial overlap can be observed between the end of the sequences.

As a consequence, read length usually exceeds the length of the DNA molecule, and the read contains both the sequence of the original molecule and also part of the adaptor sequence (see Figure 1). For paired-end reads that exceed the molecule length, both the forward and reverse reads will have the sequence of the same original molecule before showing residual adaptor sequence. Similarly, molecules that are shorter than the sum of the forward and reverse read length are expected to show identical bases at the ends of both reads since the same part of the molecule is read twice. Merging of identical sequences is also expected to reduce sequencing error due to the repeated observation of the same base. Since residual adaptor sequences in the reads interfere with mapping and assembly, it is necessary to trim reads up to the start of the original molecule.

Several algorithms have been implemented to trim adaptor sequences (see (3,4) and http://code.google.com/p/ea-utils/wiki/FastqMultx) and to merge overlapping paired-end sequences (see (5,6) and https://github.com/jstjohn/SeqPrep). However, these algorithms use cutoffs for detecting adaptors and merging reads and need to be adapted to varying rates of sequencing errors. More liberal cutoffs can lead to a greater number of false positives. Other algorithms (7–9) have been designed to merge overlapping pairs but do not provide the likelihood of seeing the adaptor at the end of both reads. Furthermore, sequencing centers often give end-users sequencing data with the adaptors already trimmed and including the likelihood of sequencing the adaptor becomes impossible or use this information to reconstruct very short molecules.

We present a new Bayesian maximum *a posteriori* trimming and merging algorithm, leeHom, that is particularly useful for ancient DNA (aDNA) and other cases where short molecules are sequenced. Instead of separating the processes of adaptor trimming and merging, leeHom considers both steps into a single probabilistic model. Briefly, leeHom computes the probability of observing the reads given a certain original molecule length and returns the most likely one. Our algorithm is highly robust to sequencing error, produces few false positives and is able to handle common sequencing problems, such as missing cycles. The algorithm was tested on a set of simulated aDNA sequences where the original molecule sequenced was known, and on Neandertal sequence data. Our results show that leeHom outperforms currently available software in speed and accuracy for both simulated and real aDNA data, and that it is suitable for processing large volumes of sequence data. It can take unaligned BAM or fastq files as input and requires the sequence of the adaptors be provided.

## MATERIALS AND METHODS

### Computation of the likelihood for a given sequence length

Our approach relies on computing the probability of observing a pair of reads assuming that the original molecule is of a certain length. A similar maximum likelihood approach for paired-end reads was used in the literature for assembling 16S rRNA or polymerase chain reaction product flanked by primer sequences using partially overlapping paired-end reads (see (7)). Apart from computing the likelihood of all possible overlapping sequence lengths, the likelihood of stemming from non-overlapping pairs is also computed, thus removing the need for hard cutoffs. Furthermore, a probabilistic prior of seeing a sequence of a certain length can be added.

Given that we have sequenced paired-end reads *r*_1_ and *r*_2_, we assume that if the original sequence was shorter than the read length, each read will have, at the end, the sequences of the adaptors: *a*_1_ and *a*_2_, respectively. We define *l*_1_ = length(*r*_1_) and *l*_2_ = length(*r*_2_). The probability of observing this data given that we assume that the original sequence was of length *i*, denotated as *P*(*r*_1_, *r*_2_, *a*_1_, *a*_2_|*i*), can be computed using the following formula:

(1) \begin{eqnarray*} &&P(a_1 \approx r_1[i-1..]) \cdot P(r_1[1..i-1] \approx \nonumber \\ &&\overline{r_2}[1..i-1]) \cdot P(a_2 \approx \overline{r_2}[i-1..]) \end{eqnarray*}

where *P* represents the probability, $\overline{r_2}$ is the reverse complement of *r*_2_, the [*i*..] and [1..*i* − 1] operators denote the suffix starting at position *i* and the prefix ending before position *i*, respectively, and where an end index greater than the start one represents an empty string. The first and last terms correspond to the probability of observing *r*_1_[*i*..] and $\overline{r_2}[i..]$ given that the templates were *a*_1_ and *a*_2_, respectively. The middle term corresponds to the probability of observing the stretches *r*_1_[1..*i* − 1] and $\overline{r_2}[1..i-1]$ given that they stemmed from a common sequence. The specific equations for those two probability functions are defined in greater detail below. This probability is computed from for every *i* ∈ 0...*l*_1_ + *l*_2_. The posterior probability of any length being *i* given the data can be described using the following expression:

(2) \begin{equation*} P(i|r_1,r_2,a_1,a_2) \propto P(r_1,r_2,a_1,a_2|i) \cdot P(i) \end{equation*}

The prior on the sequence length *i* is defined using the probability density function of the log-normal distribution given by:

(3) \begin{equation*} P(i) = \frac{1}{i \sqrt{2\pi } \sigma } e^{ - \frac{({\rm ln}(i) - \mu )^2 }{2 \sigma ^2} } \end{equation*}

**Figure 2.**
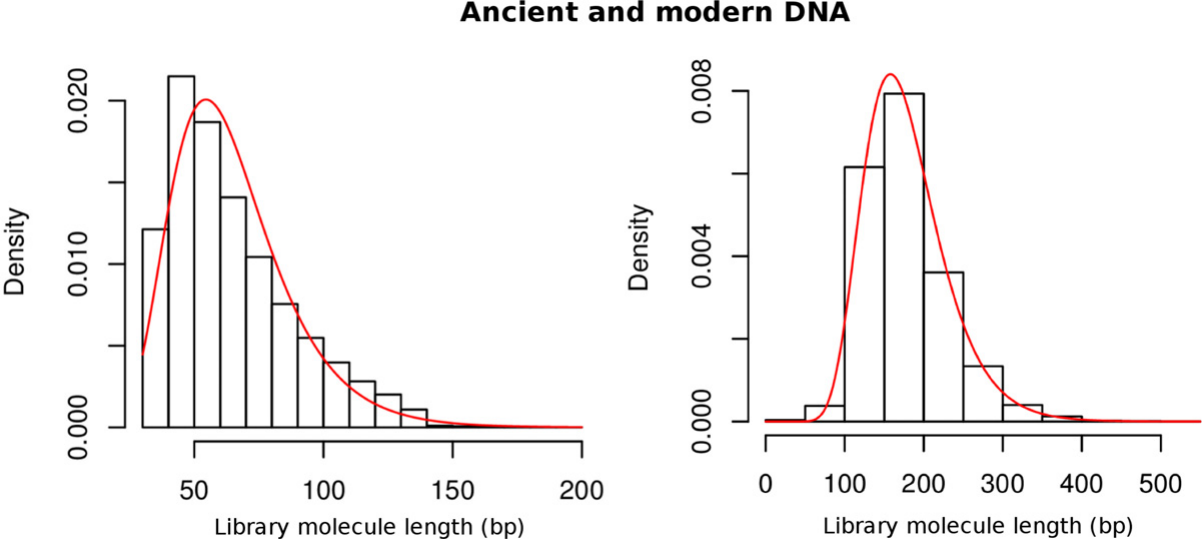
Empirical (black) and theoretical (red) length distributions of ancient and modern DNA libraries. Presented is the output of the maximum likelihood fit from the Fitdistrplus R package using a log-normal distribution for an aDNA library (left) and a modern DNA library (right). aDNA molecules tend to be of shorter length with a much narrower variance than modern DNA.

The term above models the likelihood of seeing that particular sequence size given a prior belief on the sequence size distribution. To find the most suitable distribution to model the length of DNA sequences, various heavy-tail distribution were compared using the maximum likelihood fit from the Fitdistrplus R package (http://cran.r-project.org/web/packages/fitdistrplus/) and the one maximizing the likelihood of the fit was log-normal (data not shown). To illustrate how the shape of the prior changes from modern to aDNA sequences, the log-normal distribution for both a modern and aDNA data set was computed (see Figure 2). Users also have the option of using a uniform prior on the sequence length if the size distribution of the sequences is unknown.

leeHom aims at finding the original sequence length *i*_max_ that maximize the likelihood of observation of *r*_1_ and *r*_2_:

\begin{equation*} i_{{\rm max}} = {\rm argmax}_{i \in \lbrace 0...l_1 + l_2\rbrace } P(i|r_1,r_2,a_1,a_2) \end{equation*}

and returns the most likely bases for the sequence of length *i*_max_.

To compute *P*(*a*_1_ ≈ *r*_1_[*i*..]) and *P*(*a*_2_ ≈ *r*_2_[*i*..]), we use a string comparison that disallows insertions/deletions while tolerating mismatches. The probability of seeing a substring of a read *r*[*i*..] given that an adaptor *a* was the template is given by the product of the likelihood for each base:

(4) \begin{equation*} P(a \approx r[i-1..]) = \prod _{k=i-1}^{k={\rm length}(r)} P_{{\rm match}} (a[k-i+1],r[k]) \end{equation*}

where *P*_match_ is the likelihood of match for two bases. Let *q*[*i*] be the quality score associated with base *r*[*i*], the probability of sequencing error for a given quality score *q*[*k*] is defined as follows:

(5) \begin{equation*} p_e(q[k]) = 10^{\frac{-q[k]}{10}} \end{equation*}

Therefore, the probability of observing *r*[*k* + *i*] given that the correct nucleotide is *a*[*k*] is computed as follows:

(6) \begin{equation*} P_{{\rm match}} (a[k],r[k+i]) = \left\lbrace \begin{array}{ll}1-p_e(q[k]) & \text{if } a[k] = r[k+i] \\ p_e(q_k) \cdot \frac{1}{3} & \text{if } a[k] \ne r[k+i] \\ \frac{1}{4} & \text{if } k > {\rm length}(a) \\ \end{array}\right. \end{equation*}

Equation (6) assumes that the probability of error given a certain sequenced base represents the probability of miscalling the base to any other base with equal probability.

The likelihood of the overlap $P(r_1[1..i-1] \approx \overline{r_2}[1..i-1])$, is defined as the probability of having seen both substrings given that they stemmed from the same DNA sequence. Assuming that each base is independent of the remaining ones, the likelihood for each base can therefore be multiplied as such:

(7) \begin{equation*} P(r_1[1..i-1] \approx \overline{r_2}[1..i-1]) = \prod _{k=1}^{k=i-1} P_{{\rm overlap}} ( r_1[k] , \overline{r_2}[k] ) \end{equation*}

Given that the strings *r*_1_ and $\overline{r_2}$ have the associated quality scores *q*_1_ and $\overline{q_2}$, the likelihood of two bases from two different reads stemming from the same original base is given by marginalizing the probabilities for each potential nucleotide that could have been this original nucleotide multiplied by the respective probability of observation of the two sequenced nucleotides:

(8) \begin{equation*} P_{{\rm overlap}} ( r_1[k] , \overline{r_2}[k] ) = \sum _{n \in \left\lbrace A,C,G,T \right\rbrace } P_{{\rm obs}}(n) \cdot P_{{\rm obs}}(r_1,\overline{r_2}|n) \end{equation*}

where *P*_obs_(*n*) representing the likelihood of observing nucleotide *n* in the original overlapping sequence, approximated to $\frac{1}{4}$ for ∀*n* ∈ {*A*, *C*, *G*, *T*}. The second term ($P_{{\rm obs}}(r_1,\overline{r_2}|n)$) can be quantified as follows:

(9) \begin{equation*} \left\lbrace \begin{array}{llll}(1-p_e( q_1[k] )) & \cdot & (1-p_e(q_2[k])) & \text{if } r_1[k] = \overline{r_2}[k] \wedge r_1[k] = n \\ (1-p_e( q_1[k] )) & \cdot & (p_e(q_2[k])) \cdot \frac{1}{3} & \text{if } r_1[k] \ne \overline{r_2}[k] \wedge r_1[k] = n \\ (p_e( q_1[k] )) \cdot \frac{1}{3} & \cdot & (1-p_e(q_2[k])) & \text{if } r_1[k] \ne \overline{r_2}[k] \wedge \overline{r_2}[k] = n \\ (p_e( q_1[k] )) \cdot \frac{1}{3} & \cdot & (p_e(q_2[k])) \cdot \frac{1}{3} & \text{if } r_1[k] \ne n \wedge \overline{r_2}[k] \ne n \\ \end{array}\right. \end{equation*}

Again, we assume that a sequencing error is equally likely to produce any nucleotide besides the correct one.

Once again, leeHom aims at finding the sequence length *i* that maximizes Equation (1). However, different values of *i* can be equally likely. To avoid incorrect reconstructions due to multiple sequence lengths that are equally likely, we avoid reconstructing sequences where the ratio of the likelihoods of the second most likely sequence length to the most likely one exceeds 1 in 20. This ensures that the most likely sequence has to be several fold more likely than the second-best option. As mentioned before, the likelihood of having no overlap and, therefore, having a sequence length exceeding twice the read length is also computed as follows:

(10) \begin{equation*} \int _{l_1 + l_2} ^{\infty } \frac{1}{x \sqrt{2\pi } \sigma } e^{ - \frac{({\rm ln}(x) - \mu )^2 }{2 \sigma ^2} } \cdot \prod _{l_1 + l_2} P_{{\rm obs}}(n) \end{equation*}

where *P*_obs_(*n*) is defined as in Equation (8). The prior ($\int _{l_1 + l_2} ^{\infty } \frac{1}{x \sqrt{2\pi } \sigma } e^{ - \frac{({\rm ln}(x) - \mu )^2 }{2 \sigma ^2} }$) on the sequence length represents the probability of generating a sequence longer than *l*_1_ + *l*_2_ and can be interpreted as 1 − cdf(*l*_1_ + *l*_2_), where cdf() is the cumulative distribution function for the aforementioned log-normal distribution. The resulting value is compared with the remaining likelihood values for sequence lengths.

### Consensus of overlapping regions

Once the most likely sequence length has been computed, the remaining task is to assemble the sequence using the information provided by *r*_1_ and *r*_2_. If a base has been covered in only one read, it is reported along with the original quality score. However, if the base is covered by both reads, a consensus base with its associated quality score is produced. Again, we assume a principle of independent observations with quantified error probabilities given by the quality scores to produce both quantities.

Let two sequenced bases *b*_1_ and *b*_2_ with quality scores on the PHRED scale *q*_1_ and *q*_2_, respectively. For any given nucleotide *n* ∈ {*A*, *C*, *G*, *T*} that we believe to be the actual base, the probability of observing *b*_1_ can be computed by the following:

(11) \begin{equation*} p(b_1|n) = \left\lbrace \begin{array}{@{}l@{\quad }l@{}}1-p_e(q_1) & \text{if } b_1 = n \\ \frac{p_e(q_1)}{3} & \text{if } b_1 \ne n \\ \end{array}\right. \end{equation*}

Assuming that both bases *b*_1_ and *b*_2_ represent independent observations, we can define the probability of *n* given *b*_1_ and *b*_2_:

(12) \begin{equation*} p(b_1,b_2|n) = p(b_1|n) \cdot p(b_2|n) \end{equation*}

For calling the consensus base we seek to compute the likelihood of a nucleotide *n* given the observation *b*_1_ and *b*_2_. This can be computed using Bayes’ rule:

(13) \begin{equation*} p(n|b_1,b_2) = \frac{p(b_1,b_2|n) \cdot p(n)}{p(b_1,b_2)} \end{equation*}

The probability of having observed *b*_1_ and *b*_2_ can be computed by summing the probability of having generated both bases given that we believe that they came from the same base. Since there are only four possibilities for this base, the following equation can be used:

(14) \begin{equation*} p(b_1,b_2) = \sum _{m \in \left\lbrace A,C,G,T \right\rbrace } p_{{\rm obs}}(m) \cdot p(b_1,b_2|m) \end{equation*}

where *p*_obs_(*m*) is the prior for that given nucleotide (see section above) and *p*(*b*_1_, *b*_2_|*m*) can be derived using Equations (12) and (11). In resulting BAM files, the probability of error, which is the probability of not observing *n* given the two bases *b*_1_ and *b*_2_, is reported. Hence, the following can be derived:

(15) \begin{equation*} p( - n|b_1,b_2) = 1- p(n|b_1,b_2) \end{equation*}

(16) \begin{eqnarray*} & = & 1- \frac{p(b_1,b_2|n) \cdot p(n)}{p(b_1,b_2)} \end{eqnarray*}

(17) \begin{eqnarray*} & = & \frac{ p(b_1,b_2) - p(b_1,b_2|n) \cdot p(n)}{p(b_1,b_2)} \end{eqnarray*}

By substituting the result from Equation (14) in the previous expression, *p*(−*n*|*b*_1_, *b*_2_) becomes:

(18) \begin{equation*} p( - n|b_1,b_2) = \frac{ \sum _{m \in { \left\lbrace A,C,G,T \right\rbrace \setminus n }} p(b_1,b_2|m) }{ \sum _{m \in \left\lbrace A,C,G,T \right\rbrace } p(b_1,b_2|m) }. \end{equation*}

Finally, the most likely nucleotide is produced along with its associated quality score by taking the PHRED scaled quantity defined in Equation (18).

### aDNA sequencing data

Since sequencing error rates vary between sequencing runs and even vary within a run, such a complex error rate is difficult to model and an actual data set would be needed to evaluate reconstruction accuracy. To benchmark the aforementioned programs on actual aDNA data, the first 10M reads from a paired-end Illumina HiSeq 2500 run from the Altai Neandertal (10) were used as test data set. Programs that trim and merge reads - MergeTrimReads, SeqPrep and AdaptorRemoval - were used with default parameters as comparison. The resulting reconstructed sequences were mapped back to the human reference genome (1000 Genomes version hg19) using BWA 0.5.10 (11) with default parameters. The number of aligned sequences along with the number of sequences aligning with mapping quality greater than 30 were tallied for each algorithm.

A common feature of the programs being tested is the ability to merge overlapping stretches. To evaluate whether this strategy improved sequence accuracy compared to simply trimming the adaptors and mapping both remaining reads separately, the same data set was processed by cutadapt (3) and the resulting unmerged and paired reads were mapped with BWA. The number of mismatches per aligned basepair was computed for aligned read which were merged by leeHom and simply left as trimmed paired reads by cutadapt.

## RESULTS

### Distribution of the log likelihood

**Figure 3.**
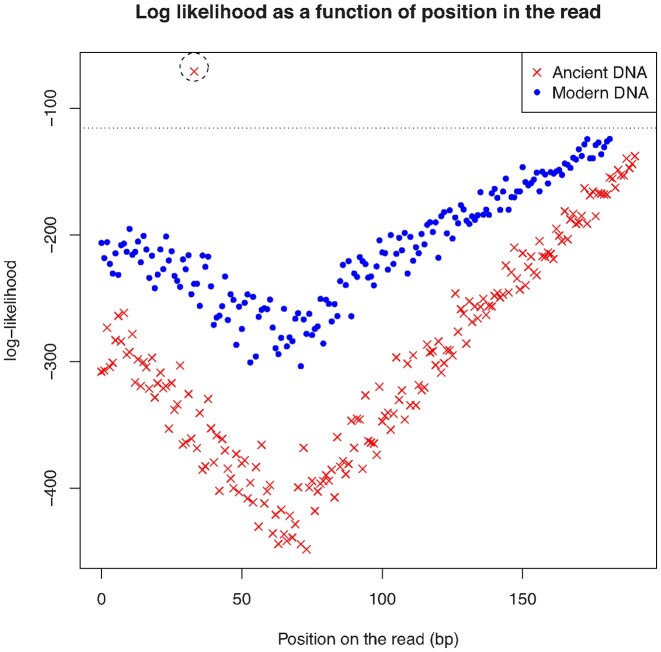
The log-likelihood for various possibilities of length of the original molecules for an ancient and modern DNA read pair. The dotted line represents the likelihood that the reads do not merge and that they came from a molecule of length greater than the longest possible overlap. For the aDNA read pairs, a particular length of the original molecule is more likely than the remaining possibilities. This is not the case for modern DNA read pairs due to the longer length of the original molecule.

To illustrate the differences in the likelihood landscape between actual modern and aDNA paired reads, the log-likelihood for different potential sequence length was plotted (see Figure 3). For aDNA pairs, there is a clear peak in log-likelihood around the length of the original fragment, whereas modern DNA shows a more even probabilistic landscape. For the aDNA, the difference between the log of the most likely and the second most likely fragment length was 66.93, whereas that difference was 0.19 for the modern DNA pairs.

### Simulated data

#### Paired-end

Using simulated paired-end reads at different levels of error, the performance of leeHom was compared to MergeTrimReads (5), SeqPrep (https://github.com/jstjohn/SeqPrep) and to AdaptorRemoval (6). Briefly, sequences matching the sequence length distribution of aDNA molecules generated for the Denisova genome project (12) were selected at random from the genome. Sequences with unresolved base pairs (‘N’) were removed. Reads of 100 bp were simulated by either adding adapter sequences to the end of reads if the original sequence was shorter than the simulated read length or by simply taking the first hundred base pairs from each end. An Illumina error profile was used by aligning PhiX control sequences to the PhiX genome and building a frequency table for matches and types of substitution. The frequency of quality scores associated with each were tallied. Errors were introduced at a certain rate and a nucleotide substitutions were added with the an associated quality score taken from the error profile. Errors were introduced for each base independently of each other. As our data set contains the original sequence, we assessed both the number of molecules for which the sequence was reconstructed perfectly, and the number of sequences with the correct length.

**Figure 4.**
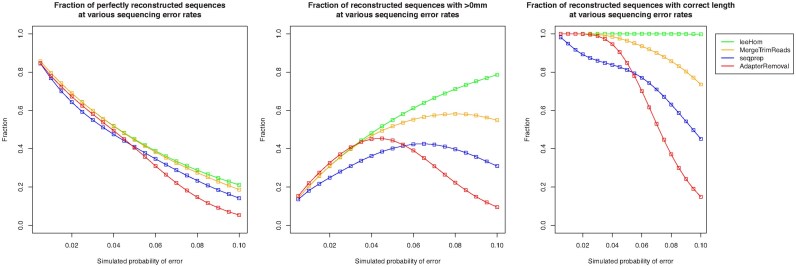
Comparison of the fraction for all input reads of reconstructed sequences as a function of simulated error rate for the output of leeHom and currently available software for sequence reconstruction based on paired-end read data. The number of perfectly reconstructed sequences (left), the ones with a single mismatch (mm) to the original sequence (center) and those with the correct length (right) are presented. Both in terms of perfectly reconstructed sequences and in terms of sequences with the correct length, leeHom outperforms other currently available algorithms.

The number of perfectly reconstructed sequences versus the simulated error rate is plotted in Figure 4. Clearly, the number of inferred sequences without any mismatches decreases both due to the increased difficulty of inferring the original sequence and the smaller number of sequences with no mismatches. The relative number of sequences with at least one mismatch was also plotted. This number tends to reach a plateau due to the absence of reads without any sequencing errors. Both in terms of perfectly reconstructed sequences and inexact matches, leeHom outperforms remaining algorithms especially at high error rates. Furthermore, in terms of reconstructed sequences with the correct length irrespective of the number of mismatches, leeHom also offers superior accuracy.

In terms of falsely merged reads, out of 931 767 paired-end reads, neither AdapterRemoval nor SeqPrep generated any false positives. MergeTrimReads and leeHom generated respectively 11 and 22 false positives. Those reads were located in regions of genomic repeats. It should also be noted that using a prior in leeHom on the sequence length equal to the distribution used to generate the simulated reads eliminates these false positives.

#### Single-end

In a similar approach to the one taken for paired-end reads, the number of perfectly inferred sequences was tallied for single-end reads for various software packages that trim adaptor sequences. Also, the number of sequences with imperfect matches to the original simulated sequence as well as the total number of sequences with correct length was computed (see Supplementary Figure S2). leeHom and AdapterRemoval offer the greatest robustness to sequencing errors. Upon measuring the amount of false positives on simulated modern DNA reads (see Supplementary Table S1), leehom offers fewer false positives than AdapterRemoval.

### Sequencing data

On an actual aDNA data set of 10M paired-end reads from (10), the runtime as well as the number of inferred sequences mapping back to the genome was computed (see Table 1). Also, the number of sequences aligning with mapping quality of at least 30 was also computed. Since an algorithm is unlikely to produce a sequence that aligns to the human genome by chance and even more unlikely to align with high mapping quality, the number of aligned sequences indicates the accuracy of the reconstruction. Both in terms of runtime and accuracy, leeHom outperforms currently available programs.

**Table 1.** Runtime and accuracy for various adapter trimming and merging software packages

	leeHom (+prior)	leeHom	MergeTrimReads	AdapterRemoval	SeqPrep	cutadapt + FLASH
runtime (wallclock)	17m16s	16m51s	60m17s	27m37s	23m20s	4m32+5m37
runtime (CPU)	17m14s	16m49s	60m16s	28m16s	24m27s	6m00+5m15
Mapped	3 381 755	3 373,531	3 370 675	3 308 763	3 222 585	3 276 250
MQ30	2 814 558	2 806 692	2 803 915	2 758 884	2 743 703	2 744 661

The runtime of different algorithms for sequence reconstruction along with the number of produced sequences aligning to the human genome. In terms of aligned sequences both at minimum mapping quality 0 and 30, leeHom outperforms other algorithms especially if a prior on the sequence length is used. Also in terms of runtime, leeHom compares favorably to other programs. PEAR failed to run due to the amount of data even when increasing the amount of RAM (to 5GB). The time reported for FLASH is the time for cutadapt to run for both forward and reverse reads and for FLASH to run.

Trivially, it should be noted that the use of any of these tools is an improvement over aligning the raw sequences without any attempt at adaptor trimming and paired-end read merging since, out of 10M paired-end reads, only 1 506 567 (15.07%) reads align and among those, 1 338 397 (13.38%) have high mapping quality.

An assumption behind paired-end read merging for aDNA is the ability to cross-correct using double observations of the sequenced bases and quality scores. To test this hypothesis, the number of mismatches per aligned base was computed for both merged sequences produced by leeHom and trimmed reads produced by cutadapt (see Table 2). The number of mismatches per aligned nucleotide is lower in the merged reads produced by leeHom thus indicating the gain in accuracy is due to cross-correction.

**Table 2.** Mismatches per aligned base for various aDNA strategies

Approach	Mismatches	Aligned bases	Mismatches per 1000 bases
leeHom	1 130 159	218 746 206	5.17
cutadapt	2 326 041	410 027 512	5.67

The number of mismatches and aligned bases for both merged sequences produced by leeHom and reads trimmed by cutadapt. This table presents the raw number of aligned nucleotides given that, for a given paired-end read, the merged sequences produced by leeHom and the trimmed sequences produced by cutadapt were aligned to the human genome. By computing the number of mismatches per nucleotide, leeHom produces sequences that have greater similarity to the human reference due to cross-correction of the reconstructed sequence using the paired reads.

## DISCUSSION

The tasks of stripping residual adapters and merging overlapping pairs are generally separated. Our results show that considering both at once in a single model increases the number of sequences that can ultimately be mapped. Furthermore, the use of the prior distribution can help distinguishing between corner cases. For instance, when a few bases of the adapters are seen, the decision to trim or not depends heavily on the prior probability for the length distribution. For very short aDNA molecules, trimming such bases might be beneficial, whereas for longer molecules, resolved bases might be needlessly removed. A Bayesian approach given the distribution offers the possibility of a natural probabilistic transition from very short fragment size to longer ones without the use of arbitrary cutoffs. A prior on the distribution should, therefore, be used whenever there is data from the same library that provides information about the size distribution of the library inserts. If no previous data on insert-size are available, the default parameters should be used.

Stricter cutoffs can be used for high quality data sets this will reduce the number of false positives. More liberal cutoffs should be used on data sets with higher errors as this will allow more sequences to be retrieved. However, as mentioned before, error rates vary between sequencing runs and often within a single sequencing run. Adapting the thresholds for the detection of the adapters and the overlapping within a single sequencing run is generally infeasible. Probabilistic approaches obviate this need by returning the most likely model given the data at hand. As shown in the results section, this approach outperforms currently available algorithms especially at high error rates.

Since adapters are often simply trimmed and the reads left unmodified during standard processing, the value of merging overlapping parts for aDNA studies was evaluated. As shown in the results section, the cross-correction effect of having observed the same sequence twice reduces noise and mismatches to the reference.

leeHom can be used with a prior on the distribution of the molecule lengths. However, this information is not always available beforehand especially for newly sequenced libraries. Ideally, the step of trimming adapters and merging overlapping parts should be combined with mapping where the distribution of the original sequences could be empirically determined. Once sufficient confidence in the shape of the fragment size distribution, this could be used as prior for both aDNA and modern samples as a standalone tool. Furthermore, substitution rates to remaining nucleotides have been assumed to be equally likely which is empirically not the case (13). More realistic substitution probabilities could be incorporated in our model. Also, leeHom assumes that quality scores are correlated positively with their observed error rate which is increasingly the case for modern sequencers.

## CONCLUSION

leeHom outperforms currently available algorithms for reconstruction of aDNA sequences from reads both in terms of accuracy and speed. The maximum likelihood sequence reconstruction lowers error in aDNA, and other data sets with overlapping paired end reads, thus leading to more accurate alignments.

## Supplementary Material

Supplementary Data
