# Supplementary material for: leeHom: adaptor trimming and merging for Illumina sequencing reads
Source: Nucleic Acids Res. 2014 Aug 6;42(18):e141. doi: 10.1093/nar/gku699 (PMC4191382; doi:10.1093/nar/gku699)
Supplement: Supplementary Data [file nar_42_18_e141_s1.zip › nar-01043-met-k-2014-File003.pdf]

SUPPLEMENTARY DATA

Sequencing routinely find that error rates differ between sequencing runs, however, quality can also differ from the location of clusters on the flowcell. To illustrate this, the expectancy of the number of mismatches for an Illumina MiSeq run with a flowcell with 2 surfaces, 2 swaths each and, in turn, 16 tiles each. The expected number of mismatches was computed for a sequence of length L using the following expression:

$$\frac{\sum_{l=1}^{L} 10^{\frac{-q_l}{10}}}{L} \qquad (1)$$

The heatmap for expected number of mismatches for each combination of surface/swath/tile was plotted (see figure 1). The error rate increases as a function of the tile number for both surfaces. Our results also show that surface 1 generally has a worse error rate than surface 2.

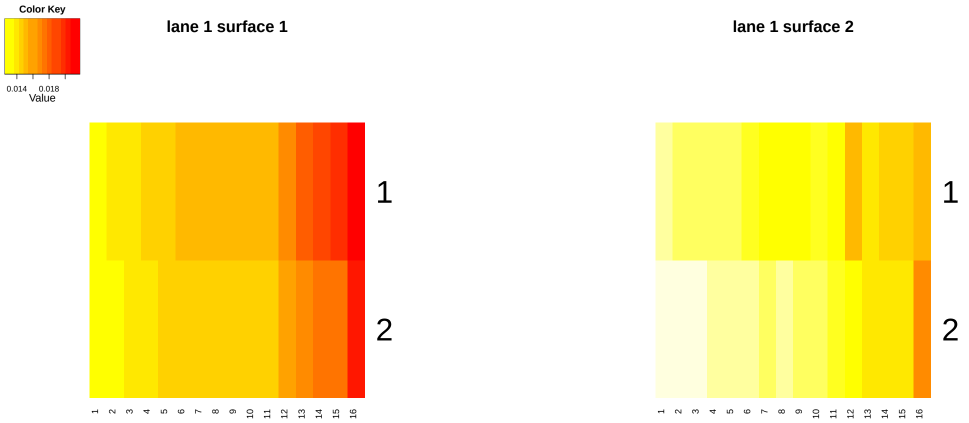

Supplementary Figure 1. Heatmap of the expected mismatch rate for an Illumina MiSeq run from 2014 with 2 surfaces, 2 swaths and 16 tiles.

As mentioned in the main manuscript, the set of forward reads for the simulated aDNA sequences were used as a test set of single-end reads. The 4 programs mentioned in the main text were used to reconstruct the original sequences (see Table 2).

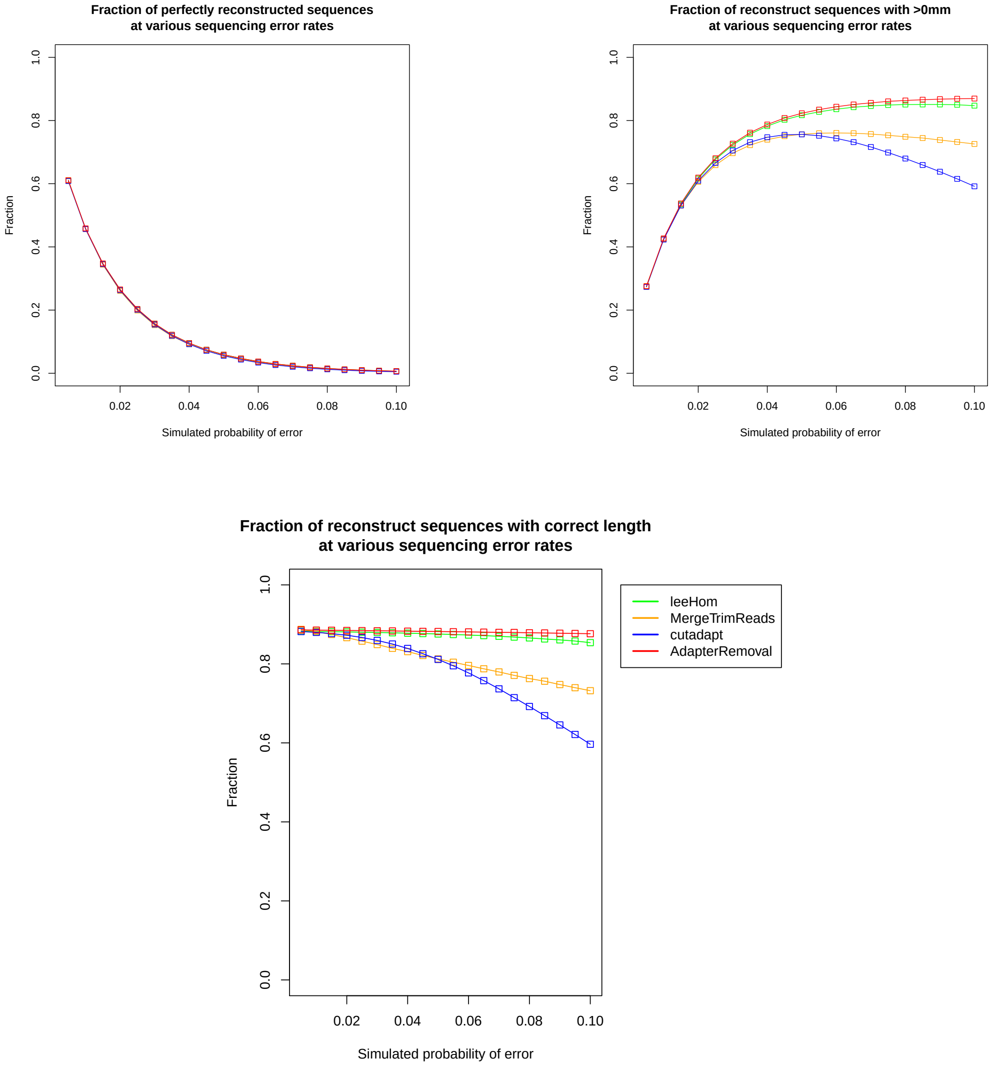

Supplementary Figure 2. Accuracy of various programs for adaptor removal on a simulated set of of single-end aDNA reads. The number of sequence with no mismatches (left), those with a single mismatch (mm) to the original sequence (center) and those with the correct length (right) are presented. leeHom and AdapterRemoval offer the most liberal trimming while cutadapt is the most conservative.

For a set of 931,767 reads taken at random from the human genome, we computed the number of sequences that were trimmed. The result reported in Table 1 show that leeHom without a prior on the sequence length generates few false positives but using a prior on the sequence length generates no false positives due to the low likelihood of observing such short sequences.

Table 1. Number of false positives for single-end reads for 931,767 reads

Software:	leeHom (prior)	leeHom	MergeTrimReads	AdapterRemoval	cutadapt
	0	102,964	359,103	164,744	25,553

Number of false positives on a simulated set of single-end modern DNA reads. A false positive is defined as any trimmed read as the simulated insert size (1000bp) should not yield any overlap with the adaptors. As mentioned in figure 2, cutadapt is the most conservative while other tools tend to trim more liberally. However, cutadapt also has lower sensitivity at higher error rates for aDNA while leeHom offers higher accuracy for aDNA reads while yielding fewer false positives than MergeTrimReads and AdapterRemoval.
